# An Interview with Cell Therapy Pioneer, Arnold Caplan

**DOI:** 10.1093/stcltm/szac026

**Published:** 2022-06-06

**Authors:** Anthony Atala

## Introduction

New technologies arise slowly through a process that has many iterations. At each iteration, a new truth, or a fact, is established and tested by the scientific community. The dogmas of 60 years ago have been sequentially tested, and new facts and new technologies provide the process of scientific advancement. Scientists who introduced new logics are often called pioneers. However, these individuals carry a heavy burden, because the truths of their initial findings and the long-term reality of that technology are often different. The healthy and vigorous interchange between the naysayers who doubt a new truth and the originators, the pioneers, is a healthy and useful process. In the long term, we in science develop new technologies that have substantial scientific impact on both old and new problems. Pioneers often stumble and are bloodied, but their perseverance has scientific, medical, and societal advantages. This interview highlights the details and complex “scientific truths” that have evolved in one person‘s scientific travels. Arnold Caplan is Professor of Biology and the Director of the Skeletal Research Center at Case Western University in Cleveland, Ohio, and a true pioneer in our field. Dr. Caplan is also the winner of the Regenerative Medicine Foundation’s 2022 Stem Cell and Regenerative Medicine Action “Lifetime Achievement” Award for important contributions in promoting scientific discovery to improve health and deliver cures.

**Figure:**
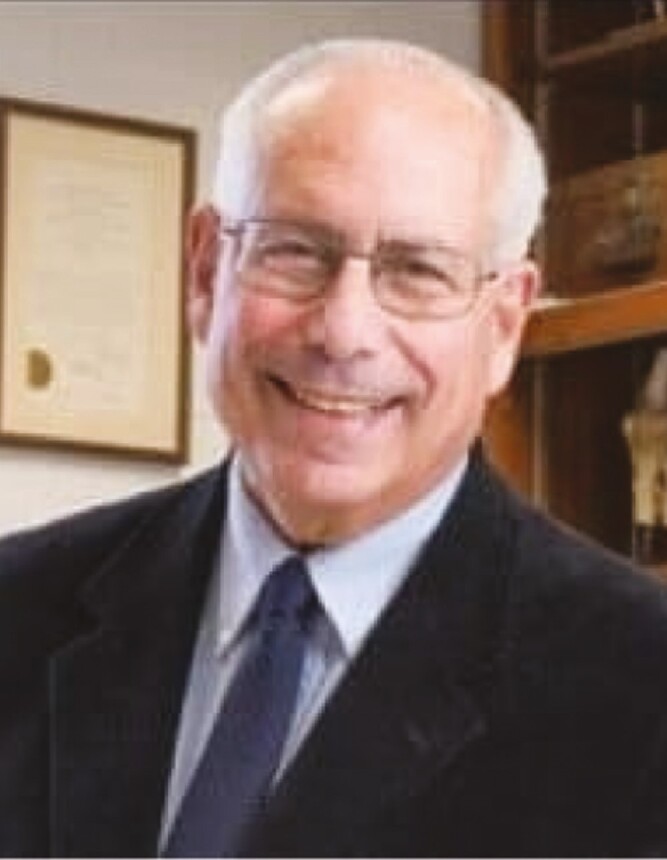
Dr. Anthony Atala

**Figure:**
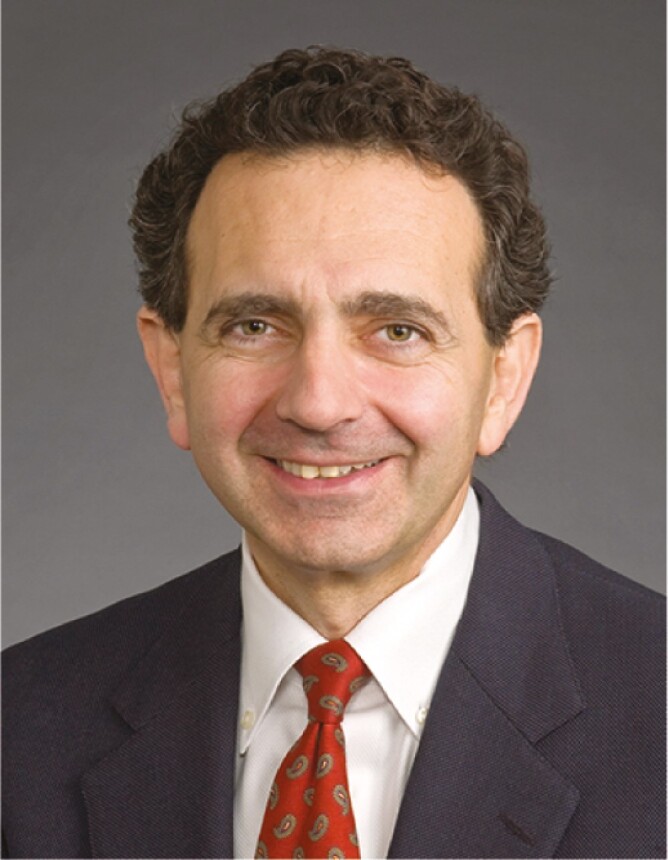
Dr. Arnold Caplan

**Anthony Atala**: At meetings where you speak, you are often introduced as a pioneer in the area of cell-based therapy and as the “father” of mesenchymal stem cells (MSCs). What is your reaction to these descriptors?

**Arnold Caplan**: The descriptor of a pioneer is a heavy burden for those of us involved in bench science, since it says that we have blazed a new trail and have established certain precedents and innovations as part of the knowledge-based progression of the biological/medical sciences. New ideas are hard to come by, and very often they are not easily accepted or within a reasonable timeframe. Likewise, new ideas are sometimes wrong but serve to stimulate vigorous attempts to disprove them and sometimes from this disproving activity, new approaches to older ideas are developed.

I recognized this burden early in my career when I was a graduate student in the laboratory of Albert Lehninger at Johns Hopkins Medical School. I sent Professor Lehninger (who was away from the laboratory on sabbatical at that time) a draft of my first ever to be published manuscript in 1965. In the Discussion section of the draft manuscript, I articulated a speculative interpretation based on the data that I had amassed. This speculative interpretation involved how calcium ions were transported into the mitochondrial interstices passing inward from the outside media eventually through the inner membrane. I proposed that the transport of calcium had nothing whatsoever to do with the oxidative phosphorylation chain, but rather was through a transporter, which was coincidental within the membrane in which the energy production system and electron transport was housed. This not only went against the dogma in Lehninger’s laboratory but also was in opposition to his published interpretation, which stated that calcium transport into the interstices of the mitochondria happened through the functioning of the electron transport chain itself.

At this time, unbeknownst to me, another hypothesis had been put forth by Professor Peter Mitchell in England, which stated that there was no electron transport chain, per se, but rather an electrical potential differential between the inside and outside of the inner mitochondrial membrane that was controlled by hydrogen ion transport across this membrane. Lehninger was in strong opposition to Mitchell’s theory which, in the end, was accepted by the biochemical community and, indeed, Peter Mitchell received a Nobel Prize for his work on the role of membrane potentials in energy production [The Nobel Prize in Chemistry 1978 was awarded to Peter D. Mitchell “for *his contribution to the understanding of biological energy transfer through the formulation of the chemiosmotic theory*.”].

The lesson for me was that my interpretation was, indeed, correct but that the local dogmas did not allow for an alternate hypothesis. For me, this slap on the wrist by Lehninger, which forced me to change the verbiage of my first publication draft, not only hurt my pride but also, in the end, was a block that impeded innovative thought and potential progress in that field of study. The other takeaway from this experience is that it will require a huge amount of time and energy in order to change the standard, accepted explanation, the dogma of the day, within a scientific context.

**Anthony Atala**: How should we in science reconcile the dogma of the day with scientific truth?

**Arnold Caplan**: By calling Lehninger’s view, “the dogma of the laboratory” is a prejudicial descriptor. What occurs is that our scientific “truth” (the dogma) of the moment may be based on putting together observations in a way which makes sense at the moment but when additional information is provided, that truth must be amended to provide the new truth of that new moment. What we take for “fact” today may not be correct tomorrow, and that’s the huge burden of the pioneer who must present the truth as they know it in that moment but must be ready to make amendments as are required by access to new information or using new technology.

Scientific fact is built on the quality and insightfulness of the observations of the moment: new technology provides a new view of the same system and often provides new insight to allow the *amended truth* of that new moment.

**Anthony Atala**: How did you end up working with “mesenchymal stem cells” (MSCs)?

**Arnold Caplan**: This is a long story and involves a process of innovation that brought me out of mitochondria into a totally different field of study. After leaving Johns Hopkins University Medical School where I got my PhD studying the inner and outer mitochondrial membranes in 1966, I accepted a postdoctoral fellowship with Professor Nathan O. Kaplan, PhD, at Brandeis University in the Department of Biochemistry where he was the chairperson. Professor Kaplan and I had agreed upon a project that involved the isoforms of the enzyme LDH and their changes in developing and repairing muscle upon innervation, or in my project’s case, upon re-innervation. I was very unhappy with this project when I started to try to set up my experimentation and so I buried myself in the library and hunted for an alternate project that would be interesting for me and for Dr. Kaplan. Professor Kaplan had previously published the effects of nicotinamide and its analogs and the formation and functioning of NAD/NADH. In the literature, I found other publications in which analogs of nicotinamide caused teratology in developing chick embryos: one group of molecules caused muscle defects and another group of molecules caused bone and cartilage defects. My naïve idea was to initially set up a culture of developing embryonic chick skeletal muscle cells as described in the 1960s by Irwin R. Konigsberg and coworkers and expose these cells to the teratogens and to unravel the biochemical mechanism of action in the myogenic developmental process in cell culture.

To accomplish these experimental objectives, Nate Kaplan introduced me to a wonderful gentleman, Professor Edgar Zwilling in the Biology Department, who agreed that I could work in his laboratory to establish this culture system and run my experiments. I did not know Zwilling from a hole in the wall and I had never read any of his classic papers in the mid-1950s involving the development of embryonic chick limb buds.

After joining Zwilling’s laboratory in 1967, I helped develop the technology to isolate the undifferentiated mesodermal cells from the developing embryonic chick limb buds. This system allowed me to study the differentiation of cartilage, bone, and muscle derived from these assumed-to-be multipotent embryonic limb bud cells in culture, which we published in 1968 in *Science*. Thus, the project that I worked on as a postdoc (1967-1969) involved the embryonic development of cartilage, bone, and muscle from the developing mesenchyme that showed that the external concentration of nicotinamide in cell culture controlled the cytoplasmic concentration of NAD, which eventually involved the PolyADP-ribosylation of histones as the cells were differentiating. This experience helped me to differentiate from a trained biochemist to become a developmental biologist who was hired in 1969 by the Biology Department of Case Western Reserve University to teach their long-standing courses in Developmental Biology and Embryology using my newly acquired expertise (thank you, Ed Zwilling).

**Anthony Atala**: How did you decide to name the cells that you first published in 1991 “mesenchymal stem cells” (MSCs)?

**Arnold Caplan**: In the late 1970s, I went to a Gordon Conference and was mesmerized by a lecture given by Professor Marshall Urist, MD, on the effects of demineralized bone and the formation of de novo new bone in the muscle pouch of adult mice. Marshall Urist was an orthopedic surgeon at UCLA with an innovative and inquisitive mind. He described demineralized bone from a variety of sources and how, by taking a piece of this demineralized bone and placing it in a muscle pouch of an adult mouse, 6 to 8 weeks later, new bone could be observed in this abnormal site. Dr. Urist deduced that inductive molecules must have leached out of the demineralized bone and stimulated progenitor cells to form bone in this abnormal site. He named these factors bone morphogenetic proteins, or BMPs, and spent many years trying to purify these molecules from demineralized bone.

In the context of trying to purify these “BMPs”, I joined the race and developed an assay for molecules found in a high-salt extract of demineralized bone. The extracted factors were placed into the medium that bathed cultures of undifferentiated embryonic limb bud cells; these extracts caused the cells to form cartilage under conditions where cartilage never usually formed (a study that we published in *Developmental Biology* in 1985). My laboratory then tried to purify these molecules and, indeed, we were competitors and in a race with my good friend Marshall Urist.

In the late 1980s, John Wozney, PhD, and colleagues at a company in Cambridge, MA, called the Genetics Institute, purified the genes that code for these proteins. These successful efforts not only validated all of Marshall Urist’s work but also identified a whole family of powerful, inductive molecules in the TGF-B family.

Having failed to win the BMP race, I was intrigued by the original Urist observation and concept that there must have been a receptive cell in the adult mouse muscle that responded to the BMPs. Steven Haynesworth, PhD (a postdoctoral fellow in my lab), and I started to purify culture adherent cells from fresh scopes of human bone marrow (known to have osteochondral progenitors) which could be expanded and induced into the cartilage and bone lineages in culture. The unique trick to this new technology was the use of the optimized culture medium (selected batches of fetal calf serum) which had been previously used with the embryonic chick limb bud cell cultures. Because of the mesodermal origin of the embryonic chick limb bud cells, I called these adherent human marrow cells *mesenchymal stem cells (MSCs),* because we could cause them to differentiate into mesenchymal phenotypes in culture, and coined the term in a publication in the *Journal of Orthopedic Research* in 1991.

**Anthony Atala**: I know from our many discussions and public lectures that you have changed your view regarding MSCs, generating in effect a new scientific “truth”.

**Arnold Caplan**: This is related to our adventures in the late 1980s and early 1990s into the MSC-world that I then assumed gave us insight into how mesodermal tissues inside the body turned-over and rejuvenated themselves. The dogma of the day was that “what you saw in culture” is what happened in the body. This dogma is now absolutely incorrect and, thus, calling these cells *stem cells* was a mistake on my part, which I tried to correct in 2010 in a publication in *Tissue Engineering*. The mistake was to aggressively disseminate this concept that the MSCs in culture represented multipotent cells that were inside the body and accounted for newly formed replacement tissue during the normal turnover and the injury-response processes in the body.

We now know that every tissue of the body, without exception, has intrinsic, tissue-specific committed progenitors that are limited in their differentiation capacity. Thus, heart progenitors can only form heart myocytes, kidney progenitors can only form cells in the kidney, and the blood cell progenitor, which has been called the hemopoietic stem cell. was not a stem cell but rather could only form the multiple phenotypes found in blood.

Furthermore, MSCs are derived from perivascular cells, and thus, MSCs can be isolated from any tissue that is vascularized. It is apparent that MSCs are not stem cells nor are they unusual, multipotent-committed progenitors. These cells are Sentinels for monitoring local microenvironment around injured or inflamed blood vessels. As Sentinels, they are guardians against the overreaction of the immune system and the infiltration of other microorganisms, especially bacteria and virus particles. This distinction, thus, defines MSCs as a unique committed phenotype that functions to both analytically survey the microenvironment and to react to stress and dysfunctional and potentially destructive local events.

It is important to emphasize that when these MSCs are culture expanded for multiple passages, their genomes are derepressed and are thus, responsive to powerful inductive agents placed in their cell culture microenvironment. The phenotypic differentiation which occurs after such sensing of the culture microenvironment is of a “sentinel activity” quite different from that of an authentic pluri- or multipotent stem cell.

**Anthony Atala**: So basically, due to new scientific insights, we need to have a paradigm shift in terms of our view of the functionality of MSCs?

**Arnold Caplan**: Correct, MSCs are not stem cells. Although I erroneously presumed that MSCs would and could provide replacement cells for those cells that were at sites of injury or sites where mature cells had naturally expired, it is clear that the MSCs are not multipotent and cannot differentiate inside the body into mesenchymal phenotypes. In addition, it is now understood that, once the MSCs are in culture and are expanded, their functionality is affected. It appears that the culturing process derepresses the genome in such a way that, exogenously added, very powerful inductive agents can stimulate these cultured cells to enter and progress down phenotypic lineage pathways. This allows cartilage, fat, and bone phenotypes to be separately observed in vitro when culture-expanded cells are exposed to the specific powerful inductive microenvironments that provide transcriptive accessibility to a variety of mesenchymal phenotypes.

Likewise, the assumption that the multipotency observed in vitro is what occurs inside the body is clearly incorrect in that this multipotency is not a part of normal functions of MSCs found within a variety of tissues. Last, as the popularity of studying MSCs increased within the scientific community, it became obvious that MSCs could be obtained from a variety of tissues. Modern single-cell RNAseq technology shows that, in comparing the cultured MSCs from human fat or MSCs from marrow, there are more than 17,000 transcripts in common, while each tissue-type MSC has 1400 to 1600 tissue-specific sequences, which characterize the MSCs derived from those tissues, respectively. Moreover, we now know that MSCs secrete huge quantities of cytokines and growth factors into the nutrient medium, and although this was overlooked initially in a publication in the *Journal of Cell Physiology* in 1996, these observations emphasize that this secretory capacity represents the primary function of these cells.

**Anthony Atala**: How would you define today the major role of MSCs?

**Arnold Caplan**: MSCs are medicinal; MSCs are derived from perivascular cells that are situated outside of and on every single blood vessel in the body. When a blood vessel is broken or inflamed, the perivascular cell, the pericyte, comes off and some of these liberated cells differentiate into MSCs. MSCs can be isolated by their culture dish adherence and expanded in number in such cultures. Such culture-expanded MSCs can be infused or delivered back into the body, where they circulate and dock at sites of injury or inflammation. Those newly docked MSCs are capable of surveying and sensing the microenvironment in which they find themselves, and they have a programmed response-profile of secretory activity for any given microenvironment. If the microenvironment is inflammatory, the MSCs produce anti-inflammatory molecules. If that microenvironment has huge amounts of bacteria, the MSC secretes inflammatory molecules to bring the monocytes and macrophage into proximity of those invading bacteria to try to rid the system and tissue of those infiltrating intruders. Thus, MSCs are site-regulated, multidrug dispensers that function at sites of injury. When culture-expanded MSCs are added to the blood system of an animal or a human, the added MSCs search for sites of tissue damage and inflammation, dock at these sites of injury, survey that site, and provide a spectrum of secretory molecules in response of that sensing activity. I say that MSCs are drugstores for sites of injury and inflammation.

**Anthony Atala**: Can you share with us your insights into the current uses of MSCs clinically?

**Arnold Caplan**: If one goes to the website clinicaltrials.gov and puts mesenchymal stem cells into their search engine, one finds well over 1,000 clinical trials that are listed for a large variety of clinical symptoms. Some of these clinical symptoms are, for example, Crohn’s disease, graft versus host disease, MS, ALS, kidney transplantation, acute myocardial infarct, heart failure, rheumatoid arthritis, lupus, autism, sepsis, and others. In many cases, phase 1 and 2 trials focusing on a particular clinical situation show that the MSCs are highly efficacious. Only a few phase 3 trials have been attempted but there are still no FDA-approved MSC products in the United States. There are 12 approved MSC products worldwide, and other phase 3 trials are now in play for clinical issues such as stroke, low back pain, and osteoarthritis. It is clear from the results of these clinical studies that the MSCs function as cytokine and growth factor secretory factories, not as stem cells.

**Anthony Atala**: Recent publications are showing benefit for COVID patients treated with MSCs. What are your overall thoughts on MSCs and COVID?

**Arnold Caplan**: MSCs can be curative for COVID-19. MSCs have been used in more than 135 clinical trials for treating patients with COVID-19 (clinicaltrials.gov). The literature clearly documents that MSCs can manage the “cytokines storm” of the immune system in SARS-CoV-2 infected patients; MSCs can facilitate tissue regeneration, including lung and vasculature; can produce molecules that can kill bacteria that massively infect the lungs of COVID-19 infected patients; and that MSCs have been used in pain management. The SARS-CoV-2 virus has an external protein, called Spike protein, which binds to a receptor on the cells’ surface, called AEC-2. After entering a cell, the virus replicates and eventually kills the cell and breaks out causing a tissue defect. If this happens in lung vasculature, one could understand how dangerous clots can form. MSCs produce molecules that can bind to either or both the Spike proteins and/or the AEC-2 receptors; such MSC-produced proteins can eliminate the virus particles from their aggressive replication cycle.

In Beijing, China, at the YouAn Hospital, from January 23 to February 16, 2020, (long before we knew that the SARS-CoV-2 virus was already in the U.S.) seven critically ill COVID-19 patients were assessed for 14 days following infusion of allogeneic, marrow-derived, and culture-expanded MSCs. This infusion of MSCs cured or significantly improved the functional outcomes of these seven severely infected patients without any adverse events. The pulmonary function and symptoms were significantly improved in 2 days after MSC transplantation. After treatment, the peripheral lymphocytes were increased, C-reactive proteins decreased, and the over-activated cytokine-secreting immune cells CXCR3+CD4+ T cells, CXCR3+CD8+ T cells, and CXCR3+ NK cells disappeared in 3-6 days. In addition, a group of CD14+CD11c+CD11bmid regulatory dendritic cells (DC) cell population dramatically increased. Meanwhile, the level of TNF-alpha was significantly decreased, while IL-10 increased in MSC-treated patients compared with the placebo group. This is the first documented therapeutic application of allogeneic, culture-expanded MSCs for COVID-19 patients. The positive therapeutic effects of MSCs in other phase 1 and 2 clinical trials in treating COVID-19 patients have recently been published as a meta-analysis by W. Qu et al.

**Anthony Atala**: Have you been involved in research related to MSCs and COVID?

**Arnold Caplan**: Since the amino acid sequence of Spike protein and the antiviral proteins that are produced by MSCs are known, my colleagues and I conducted computer simulations of binding and could document that the antibacterial/antiviral protein LL-37 could bind strongly and specially to the Spike protein and to the AEC-2 receptor. In addition, a manuscript from other investigators documenting these same observations was published in 2021 by Wang et al in *ACS Infectious Diseases*. From the above, one could assert that allogeneic, culture-expanded MSCs have the capability of being curative for severely infected COVID-19 patients.

**Anthony Atala**: Based on all the current data available on the use of MSCs for so many different applications, how would you summarize your thoughts in this area?

**Arnold Caplan**: I would have to say that MSCs are not stem cells, but they naturally have a profound functionality as Sentinels of injury and tissue disbalance. There are well over 70,000 publications on MSCs (PubMed, mesenchymal stem cells) and more recently, the medicinal capacity of MSCs has been emphasized in such publications. This new data and the use of new technologies such as single-cell RNAseq have focused on both the differences and common features of MSCs. It makes perfect biologic sense that cultured MSCs from a single source, such as fat or marrow, are heterogeneous populations. The pericytes from the vasculature deep inside of fatty tissue and those from the outside have come from different microenvironments and these different environments provide different surface chemistries to the cells that are released from these tissue sites. Which of these different MSCs are therapeutic or are they all required to bring about a therapeutic endpoint? Moreover, MSCs do not stay at a tissue site very long after they have been added to the body, but therapeutic effects can be observed for many months after the initial MSC exposure. It is clear that MSCs can be instructive to monocytes and macrophages and can cause the generation of T-regulatory cells that stay at tissue sites for years. Importantly, for example, CAR-T cells that were exogenously manipulated and then reintroduced into the donor’s body, can be found in cancer-free patients even after 10 years have elapsed, as recently published in *Nature* by Carl June’s group. Cell-based therapy such as CAR-T can be curative for some cancers, and MSCs can be curative for severe COVID-19 infections by molecular mechanisms that have been described. Neither CAR-T nor MSCs are stem cells but rather are medicinal cells that make therapeutic agents or stimulate the production of therapeutic cells at the right place at the right time.

**Anthony Atala**: Any final words of wisdom for our readers?

**Arnold Caplan**: Cell-based therapy is in its infancy and those of us who have helped to nurture it have accepted to modify our scientific truths as more information becomes available. The naysayers of cell-based therapy have done the medical profession and the patients a great service by stimulating us to continue to refine these potent therapeutics, as I have recently pointed out in 2019 in *Tissue Engineering*. This has allowed the dogma of the day to be modified to allow more efficacious use of cell-based therapies.

